# Marine Hospital Service Examination

**Published:** 1898-10

**Authors:** 

**Affiliations:** Treasury Department, Office Supervising Surgeon-General, Marine Hospital Service; Washington, D. C.


### Marine Hospital Service Examination.

TREASURY DEPARTMENT,
OFFICE SUPERVISING SURGEON-GENERAL,
MARINE HOSPITAL SERVICE.
WASHINGTON, D. C., August 25, 1898.

A board of officers will be convened at Washington, Wednesday, November 9, 1898, for the purpose of examining candidates for admission to the grade of Assistant Surgeon in the United States Marine Hospital Service. It is desired that applications for this examination be made before November 1st.

Candidates must be between twenty-one and thirty years of age, graduates of a respectable medical college, and must furnish testimonials from responsible persons as to character.

The following is the usual order of the examination: 1. Physical. 2. Written. 3. Oral. 4. Clinical.

In addition to the physical examination, candidates are required to certify that they believe themselves free from any ailment which would disqualify for service in any climate.

The examinations are chiefly in writing, and begin with a short autobiography by the candidate. The remainder of the written exercise consists in examination on the various branches of medicine, surgery and hygiene.

The oral examination includes subjects of preliminary education, history, literature and natural sciences.

The clinical examination is conducted at a hospital, and when practicable candidates are required to perform surgical operations on the cadaver.

Successful candidates will be numbered according to their attainments on examination, and will be commissioned in the same order as vacancies occur.

Upon appointment the young officers are, as a rule, first assigned to duty at one of the large marine hospitals, as at Boston, New York, New Orleans, Chicago or San Francisco.

After five years' service assistant surgeons are entitled to examinations for promotions to the grade of passed assistant surgeon.

Promotion to the grade of surgeon is made according to seniority, and after due examination, as vacancies occur in that grade. Assistant surgeons receive sixteen hundred dollars, passed assistant surgeons two thousand dollars, and surgeons twenty-five hundred dollars per year. When quarters are not provided, commutation at the rate of thirty, forty or fifty dollars a month, according to grade, is allowed.

All grades above that of assistant surgeon receive longevity pay, ten per centum in addition to the regular salary for every five years service up to forty per centum after twenty years service.

The tenure of office is permanent. Officers traveling under orders are allowed actual traveling expenses. For further information or for invitation to appear before the board of examiners, address, SUPERVISING SURGEON-GENERAL,

U. S. Marine Hospital Service.

